# TreeHub: a comprehensive dataset of phylogenetic trees

**DOI:** 10.1038/s41597-025-05282-4

**Published:** 2025-06-02

**Authors:** Ping Wu, Yawei Cao, Jiajie Yang, Hui Wu

**Affiliations:** 1 College of Life Science, Sichuan Normal University, Chengdu, Sichuan 610101 China; 2 Big Data and AI Biodiversity Conservation Research Center, Institute of Botany, Chinese Academy of Sciences, Beijing, 100093 China; 3 Plant Science Data Center, Chinese Academy of Sciences, Beijing, 100093 China; 4 China National Botanical Garden, Beijing, 100093 China; 5 State Key Laboratory of Plant Diversity and Specialty Crops and Key Laboratory of Systematic and Evolutionary Botany, Institute of Botany, Chinese Academy of Sciences, Beijing, 100093 China

**Keywords:** Plant evolution, Taxonomy

## Abstract

Phylogenetic relationships are crucial for solving various biological questions, serving as a fundamental knowledge in biology. However, the application of phylogenetic trees has been limited by inadequate coverage of updated published phylogenies and the scarcity of reliable comprehensive datasets. In this study, we present a novel approach for automatically extracting phylogenetic data and integrating relevant species information from scientific papers and public databases. On this basis, we constructed a dataset TreeHub, including 135,502 corresponding phylogenetic trees from 7,879 phylogenetic research articles across 609 academic journals. This database will serve as a reliable and accessible resource for the scientific community, accelerating innovations in biodiversity studies and evolutionary theory based on high-density data.

## Background & Summary

Phylogenetic trees are tree-shaped diagrams that illustrate the evolutionary relationships between species or populations^1^. The tree of life is crucial for addressing various biological questions and serves as fundamental knowledge in biology^2–4^. It is also a core tool that can integrate different types of data based on interspecies relationships, enabling cross-disciplinary research^5^. Constructing accurate phylogenetic trees is computationally intensive and laborious process. It involves extensive sequence data or morphological traits collection across various species or groups, subsequent analysis relying on numerous mathematical models, and substantial computational demands^6–8^. Additionally, the accuracy and correctness of the resulting trees depend on careful evaluation and revision^9^. With advancements in sequencing technologies, phylogenetic trees have evolved to a new level of “Phylogenomics” involving numerous genes and mathematical models^10,11^. Nowadays, a basic understanding of phylogenetic inference is essential for all biologists. It’s important to efficiently discover and adeptly manipulate phylogenetic data, rather than starting from the original sequencing data.

Phylogenetic trees, as an integral part of research findings, are often made publicly available. It is meaningful to construct repositories of phylogenetic trees and associated data matrices for easy access and developing tools. It will also accelerate innovations in biodiversity studies and evolutionary theories based on high-density data^12–14^. Besides, large-scale databases are critical for the development of novel bioinformatics software and algorithms, enhancing the precision and efficiency of existing phylogenetic analysis methodologies^15–18^. Over the past 20 years, phylogenetic databases have been developed to offer valuable services for storing and managing phylogenetic tree data, such as TreeBASE and Open Tree of Life^19,20^. These databases often relied rely on voluntary uploads from researchers, leading to information loss and delays. The website information of TreeBASE indicates that the databases have been updated to 2019. In contrast, there has been a significant emergence of phylogenetic studies, particularly those involving phylogenomic analyses^21,22^.

Therefore, the development of a comprehensive tree dataset encompassing a wide range of taxa, coupled with reliable species information, holds significant value for evolutionary biology and related fields. Beyond user submissions, web scraping and text mining offer promising alternatives for automatically collecting phylogenetic data^23–25^. However, there are challenges in retrieving phylogenetic trees from the literature. Phylogenetic tree data is often stored in various formats across different websites, ranging from compressed binary files to plain text files and specialized formats. Additionally, related species descriptions lack metadata standards and are scattered throughout the text. These inconsistencies hinder efficient and reliable data aggregation.

**Fig. 1:**
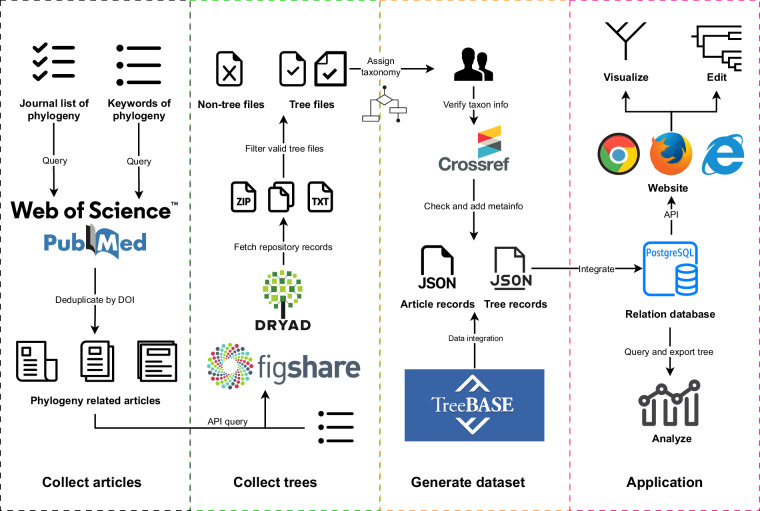
Workflow of automatically extracting phylogenetic data and its sharing platform, including phylogenetic research collection, phylogenetics trees collection, taxonomic assignment and the application for the dataset.

This research introduces a novel method for automatically extracting phylogenetic data and intregrating relevant species information from scientific papers and public databases (Fig. 1). Using this method, we curated a dataset named “TreeHub” (Fig. 1). TreeHub integrates metadata from scientific articles, their corresponding phylogenetic trees, and raw tree files, enhancing accessibility and usability for researchers. Our study provides a comprehensive resource valuable to the broad scientific community, especially supporting research in areas like evolutionary theory, macroevolution, taxonomy, bioinformatics and ecology.

## Methods

### Phylogenetic research collection

Phylogenetic trees are extensively applied across diverse fields, including Evolutionary Biology, Phylogenetics, Ecology, Paleobiology, Botany, and Zoology. To ensure comprehensive data collection, we targeted research articles related to phylogenetic trees published up to the end of January 2025. We curated a list of journals that frequently publish research on Phylogenetics and Evolutionary Biology (Supplementary Table 1), and conducted searches in two commonly used article databases, NCBI PubMed and Web of Science^26,27^. On this basis, we performed searches in other journals using a set of keywords, including “phylogeny”, “phylogenetics”, “evolution” and “systematics”. All search results were converted into JSON format to facilitate subsequent analysis and processing. Each record contained essential metadata, such as title, authors, abstract, journal name, publication date and DOI (digital object identifier) number. Finally, we utilized DOI numbers as unique identifiers to deduplicate the data.

### Phylogenetics trees collection. (1) data acquisition

We downloaded open access phylogenetic tree data from Dryad (https://datadryad.org, CC0 license) and FigShare (https://figshare.com, CC0 or CC-BY license). On the Dryad platform, we collaborated with the website administrators to secure an API key for acquiring an access token from “https://datadryad.org/oauth/token”, thereby ensuring adherence to data extraction guidelines^28^. The Search API (https://datadryad.org/api/v2/search; https://api.figshare.com/v2/articles/search) was used to retrieve records using the aforementioned DOIs as parameters. If a record contained a non-empty “identifier” (Dryad’s unique record number) field and “storageSize” (file size) in the Dryad dataset, or a non-empty “files” field in the FigShare dataset, we used the Download API (https://datadryad.org/api/v2/datasets/{identifier}/download; https://api.figshare.com/v2/file/download/) with the “identifer” value to retrieve the dataset associated with the record. **(2) Data Cleaning**. Before analyzing the file content, we checked the size of each downloaded file and extracted all compressed files as necessary. Non-compressed files were then assessed based on their filename suffix (ignoring case) to identify potential phylogenetic tree files. Valid suffixes included “.nwk”, “.newick”, “.nex”, “.nexus”, “.tre”, “.tree”, “.treefile”, and “.txt”. Subsequently, we utilized DendroPy, a Python library, to verify the files as Newick and NEXUS format, which are prevalent for representing phylogenetic trees^29^. Validated phylogenetic tree files were paired with their corresponding publication information from our initial data collection phase. These merged records were then converted into JSON format for ease of processing and analysis.

During this step, we observed that some file records within the Dryad repository contained phylogenetic trees but lacked the corresponding publication details. These records were mainly submitted just before the publication of the related paper or were not associated with journal papers. We used Dryad’s Search API, incorporating phylogenetics-related keywords as search parameters, to gather these records and performed deduplication using Dryad’s unique identifiers. To enrich these records with missing information, we utilized the Crossref API (https://api.crossref.org) to obtain complete publication metadata associated with the DOIs.

### Taxonomic assignment

Accurate taxonomic information is crucial for effective querying and meta-analysis of phylogenetic trees, which are distributed in article titles, abstracts, and tree diagram documents. This allows users to quickly retrieve data relevant to specific taxa of interest. We implemented two approaches for taxonomic assignment: one utilizing the metadata of the publications and another derived from the phylogenetic trees themselves.

Firstly, we downloaded the latest NCBI Taxonomy database (January 2025) and extracted valid taxonomic names at four ranks: order, family, genus, and species^30^. This resulted in four distinct sets: Set_Order, Set_Family, Set_Genus, and Set_Species. Secondly, for the method based on publication metadata, we tokenized the titles and abstracts of associated publications using regular expressions with spaces and punctuation as delimiters. This yielded a comprehensive set of unique words (Set_Paper). This set was then augmented with binomial nomenclature terms (capitalized genus name + specific epithet) extracted from these texts. To reduce noise and improve processing efficiency, we removed the 1000 most common English words (sourced from https://1000mostcommonwords.com/1000-most-common-english-words/) from Set_Paper, resulting in a refined set (Set_Paper2). We intersected Set_Paper2 with our taxonomic rank sets. Non-empty results from these intersections were considered candidate taxonomic names (Name1). Thirdly, for the method based on phylogenetic trees, we extracted the terminal node labels from phylogenetic tree files to create a set of sample names (Set_Sample_Name). Similar to the metadata approach, we intersected Set_Sample_Name with our taxonomic rank sets. Duplicate names within the results were allowed. The candidate taxonomic name with the highest frequency of occurrence was selected (Name2). Finally, two candidate names (Name1 and Name2) were compared to determine the final taxonomic name for the publication: a) identical names, which indicated a highly reliable result; b) different names, Name1 was preferred as the sample names in the tree files can be abbreviated, potentially leading to lower accuracy; c) undetermined, if neither method yielded a credible name, the record was marked as undetermined and reserved for manual analysis.

### Public database integration

As TreeBASE is a well-known repository for phylogenetic trees, we integrated data from this platform into our existing dataset through the following steps. Firstly, we acquired the most recent TreeBASE dataset (June 2019, CC0 license) via the TreeBASEdmp tool^31,32^. This data was then imported into a local PostgreSQL database for structured storage and analysis. Given that TreeBASE stores phylogenetic trees as nodes and links, we utilized the ‘write_trees.pl’ script to reconstruct the original tree structures from this node information. Leveraging SQL queries, we extracted metadata associated with each phylogenetic tree record from various tables, allowing us to link tree files with the corresponding metadata and publication details. Next, we employed SQL’s COPY command to export the extracted data in a JSON format, aligning with the structure of our existing crawled dataset. Finally, using DOI as a unique identifier, we deduplicated the TreeBASE data against our previously crawled dataset, ensuring a comprehensive and non-redundant collection.

## Data Records

The dataset is available at SciDB^33,34^ under CC-BY 4.0 license, or the dataset could be easy querying and retrieval at the website “https://www.plantplus.cn/treehub”. It includes 135,502 phylogenetic trees derived from 7,879 research articles across 609 journals and independent studies. The records spans a wide range of taxa, including archaea, bacteria, fungi, viruses, animals (metazoa) and plants.

**Fig. 2:**
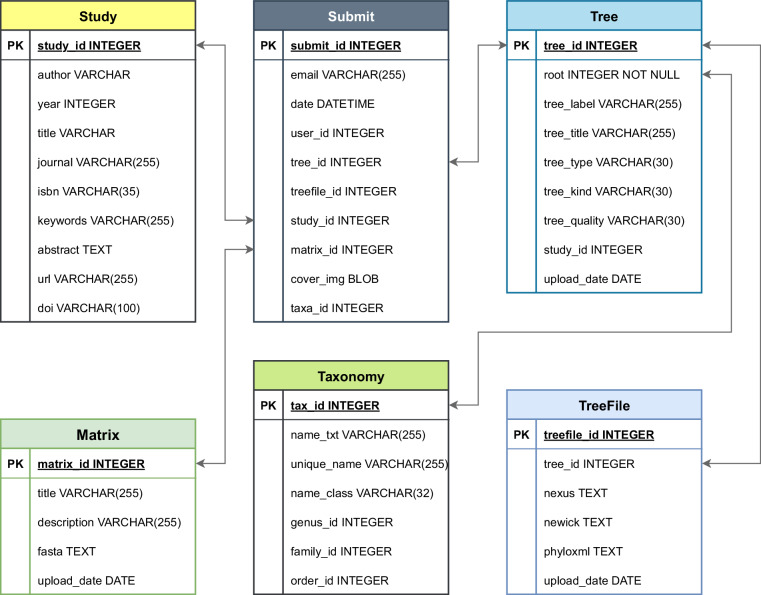
Structure of the database of TreeHub. The database includes six main tables and other auxiliary tables. PK represents the primary key of each table, which is the unique number of each record in the table. The tables are one-to-one associated through primary keys.

The structure of the database is shown in Fig. 2, and it mainly includes phylogenetic trees (table Tree and TreeFile), related articles (table Study), taxon information (table Taxonomy), sequence alignments (table Matrix), and submission or crawling information (table Submit). Field descriptions for each table are provided in Supplementary Table 2. Most fields are consistent with those in the JSON-ZIP dataset, with the exception of those in the Submit table, which are specific to the database.

TreeHub offers flexibility for users by providing data in two formats: JSON-format data with compressed tree files, and a PostgreSQL database backup. The database backup can be directly import into PostgreSQL (14.0 or newer). The JSON-format dataset contains the following JSON or ZIP-compressed files: (1) Paper.json: This file contains metadata for phylogeny-related publications in standard JSON format. Each record is uniquely identified by the publication’s DOI number, and the corresponding phylogenetic tree files are listed in the “tree_files” field.” (2) Tree.json: This compressed archive contains phylogenetic trees in Newick format. The filenames within the archive correspond to the phylogenetic tree IDs referenced in the JSON files. (3) Tree.zip: The file compressed archive containing phylogenetic trees in Newick format, and the filenames correspond to the phylogenetic tree IDs referenced in other JSON files.

## Technical Validation

To ensure the reliability of the taxonomic or species, we engaged experts in evolutionary biology to review the taxonomic information for each publication. These experts were given links to the publications and were tasked with evaluating the assigned taxonomy, including cases where the algorithm was unable to make an assignment.

The results indicated that when two methods assigned same taxonomy name for a record, the accurate rate of the taxonomic name was 100%. For names determined solely based on publication information, the accuracy rate was 95.8%, with errors mainly arising from ambiguous genus names. For example, *Mya* refers to a genus of saltwater clams but also stands for an abbreviation for “Million years” in a paper about butterflies^35^. Surprisingly, taxonomic names identified exclusively from phylogenetic trees had a low accuracy rate of 7.7%, often identifying a lower taxonomic level within the actual taxon (e.g., assigning “*Leptotila*” when the correct taxon was the broader class Aves)^36^. Besides, the program failed to assign taxonomic names to 20.9% of the papers due to several factors: the papers encompassing diverse organisms across multiple kingdoms; some studies utilized simulated phylogenetic trees; certain trees lacked identifiable species or taxonomic names. All erroneous, partially correct, or missing taxonomic information was carefully reviewed and corrected by experts through a thorough examination of the full text of each publication. Consequently, the dataset presented in this study is a verified version.

## Usage Notes

Whether accessing the dataset via JSON or the database, we recommend users employ scientific names (rather than common names) for taxon queries. For users working with the JSON-ZIP dataset and performing complex downstream analyses, we recommend parsing text records using UTF-8 encoding for enhanced internationalization support. Database users are encouraged to utilize pgAdmin (https://www.pgadmin.org/) as a client for simplified data querying and export.

## Supplementary information

Supplementary Table 1-Phylogneny related journals

Supplementary Table 2-Descriptions of fields in each table of TreeHub

## Data Availability

All the code used to generate this dataset is openly accessible on GitHub: https://github.com/wpwupingwp/tree_crawler. The scripts are written in Python and are designed to be cross-platform compatible.

## References

[1] Yang, Z. & Rannala, B. Molecular phylogenetics: principles and practice.* Nat. Rev. Genet. ***13**, 303–314, 10.1038/nrg3186 (2012). 22456349 10.1038/nrg3186

[2] Dunn, C. W., Giribet, G., Edgecombe, G. D. & Hejnol, A. Animal phylogeny and its evolutionary implications. *Annu. Rev. Ecol. Evol. Syst.* **45**, 371–395, 10.1146/annurev-ecolsys-120213-091627 (2014).

[3] Group, A. P. *et al*. An update of the Angiosperm Phylogeny Group classification for the orders and families of flowering plants: APG IV. *Bot. J. Linn. Soc.* **181**, 1–20, 10.1111/boj.12385 (2016).

[4] Pester, M., Schleper, C. & Wagner, M. The Thaumarchaeota: an emerging view of their phylogeny and ecophysiology. *Curr. Opin. Microbiol.* **14**, 300–306, 10.1016/j.mib.2011.04.007 (2011). 21546306 10.1016/j.mib.2011.04.007 PMC3126993

[5] Sandall, E. L. *et al*. A globally integrated structure of taxonomy to support biodiversity science and conservation. *Trends Ecol. Evol.* **38**, 1143–1153, 10.1016/j.tree.2023.08.004 (2023). 37684131 10.1016/j.tree.2023.08.004

[6] Larget, B. & Simon, D. L. Markov Chain Monte Carlo algorithms for the Bayesian analysis of phylogenetic trees. *Mol. Biol. Evol.* **16**, 750–759, 10.1093/oxfordjournals.molbev.a026160 (1999).

[7] Saitou, N. & Nei, M. The neighbor-joining method: a new method for reconstructing phylogenetic trees. *Mol. Biol. Evol.* **4**, 406–425, 10.1093/oxfordjournals.molbev.a040454 (1987). 3447015 10.1093/oxfordjournals.molbev.a040454

[8] Yang, Z. PAML: a program package for phylogenetic analysis by maximum likelihood. *Comput. Appl. Biosci.* **13**, 555–556, 10.1093/bioinformatics/13.5.555 (1997). 9367129 10.1093/bioinformatics/13.5.555

[9] Ran, J. H., Shen, T. T., Wang, M. M. & Wang, X. Q. Phylogenomics resolves the deep phylogeny of seed plants and indicates partial convergent or homoplastic evolution between Gnetales and angiosperms. *Proc. R. Soc. B* **285**, 10.1098/rspb.2018.1012 (2018). 10.1098/rspb.2018.1012 PMC6030518 29925623

[10] Kapli, P., Yang, Z. & Telford, M. J. Phylogenetic tree building in the genomic age. *Nat. Rev. Genet.* **21**, 428–444, 10.1038/s41576-020-0233-0 (2020). 32424311 10.1038/s41576-020-0233-0

[11] Xia, X. M. *et al*. Spatiotemporal evolution of the global species diversity of Rhododendron. *Mol. Biol. Evol*. **39**, 10.1093/molbev/msab314 (2022). 10.1093/molbev/msab314 PMC8760938 34718707

[12] Chamberlain, S. A. *et al*. Does phylogeny matter? Assessing the impact of phylogenetic information in ecological meta‐analysis. *Ecol. Lett.* **15**, 627–636, 10.1111/j.1461-0248.2012.01776.x (2012). 22487445 10.1111/j.1461-0248.2012.01776.x

[13] Healy, K., Ezard, T. H. G., Jones, O. R., Salguero-Gómez, R. & Buckley, Y. M. Animal life history is shaped by the pace of life and the distribution of age-specific mortality and reproduction. *Nat. Ecol. Evol.* **3**, 1217–1224, 10.1038/s41559-019-0938-7 (2019). 31285573 10.1038/s41559-019-0938-7

[14] Li, H.-T. *et al*. Origin of angiosperms and the puzzle of the Jurassic gap. *Nat. Plants* **5**, 461–470, 10.1038/s41477-019-0421-0 (2019). 31061536 10.1038/s41477-019-0421-0

[15] D, A. S. & Klaere, S. How well does your phylogenetic model fit your data? *Syst. Biol.* **68**, 157–167, 10.1093/sysbio/syy066 (2019). 30329125 10.1093/sysbio/syy066

[16] Di Franco, A., Poujol, R., Baurain, D. & Philippe, H. Evaluating the usefulness of alignment filtering methods to reduce the impact of errors on evolutionary inferences. *BMC Evol. Biol.* **19**, 21, 10.1186/s12862-019-1350-2 (2019). 30634908 10.1186/s12862-019-1350-2 PMC6330419

[17] Jantzen, J. R. *et al*. Effects of taxon sampling and tree reconstruction methods on phylodiversity metrics. *Ecol. Evol.* **9**, 9479–9499, 10.1002/ece3.5425 (2019). 31534670 10.1002/ece3.5425 PMC6745870

[18] Walker, J. F., Walker-Hale, N., Vargas, O. M., Larson, D. A. & Stull, G. W. Characterizing gene tree conflict in plastome-inferred phylogenies. *PeerJ* **7**, e7747, 10.7717/peerj.7747 (2019). 31579615 10.7717/peerj.7747 PMC6764362

[19] Vos, R., Lapp, H., Piel, W. & Tannen, V. TreeBASE2: Rise of the machines. *Nat. Preced.*10.1038/npre.2010.4600.1 (2010).

[20] Hinchliff, C. E. *et al*. Synthesis of phylogeny and taxonomy into a comprehensive tree of life. *Proc. Natl. Acad. Sci. U.S.A.* **112**, 12764–12769, 10.1073/pnas.1423041112 (2015). 26385966 10.1073/pnas.1423041112 PMC4611642

[21] Wei, Z. R., Jiao, D., Wehenkel, C. A., Wei, X. X. & Wang, X. Q. Phylotranscriptomic and ecological analyses reveal the evolution and morphological adaptation of *Abies*. *J. Integr. Plant Biol.* **66**, 2664–2682, 10.1111/jipb.13760 (2024). 39152659 10.1111/jipb.13760

[22] Wan, J. N. *et al*. The rise of baobab trees in Madagascar. *Nature* **629**, 1091–1099, 10.1038/s41586-024-07447-4 (2024). 38750363 10.1038/s41586-024-07447-4 PMC11136661

[23] Al-Daihani, S. M. & Abrahams, A. A text mining analysis of academic libraries’ tweets. *The Journal of Academic Librarianship* **42**, 135–143, 10.1016/j.acalib.2015.12.014 (2016).

[24] Hassani, H., Beneki, C., Unger, S., Mazinani, M. T. & Yeganegi, M. R. Text mining in big data analytics. *Big Data Cogn. Comput.* **4**, 1, 10.3390/bdcc4010001 (2020).

[25] Lee, J. *et al*. BioBERT: a pre-trained biomedical language representation model for biomedical text mining. *Bioinformatics* **36**, 1234–1240, 10.1093/bioinformatics/btz682 (2020). 31501885 10.1093/bioinformatics/btz682 PMC7703786

[26] Birkle, C., Pendlebury, D. A., Schnell, J. & Adams, J. Web of science as a data source for research on scientific and scholarly activity. *Quant. Sci. Stud.* **1**, 363–376, 10.1162/qss_a_00018 (2020).

[27] Williamson, P. O. & Minter, C. I. Exploring PubMed as a reliable resource for scholarly communications services. *J. Med. Libr. Assoc.* **107**, 16, 10.5195/jmla.2019.433 (2019). 30598645 10.5195/jmla.2019.433 PMC6300231

[28] Vision, T. The Dryad Digital Repository: Published evolutionary data as part of the greater data ecosystem. *Nat. Preced.*, 1-1, 10.1038/npre.2010.4595.1 (2010).

[29] Sukumaran, J. & Holder, M. T. DendroPy: a python library for phylogenetic computing. *Bioinformatics* **26**, 1569–1571, 10.1093/bioinformatics/btq228 (2010). 20421198 10.1093/bioinformatics/btq228

[30] Schoch, C. L. *et al*. NCBI Taxonomy: a comprehensive update on curation, resources and tools. *Database* **2020**, baaa062, 10.1093/database/baaa062 (2020). 32761142 10.1093/database/baaa062 PMC7408187

[31] Conrad, A. Database of the Year: Postgres. *IEEE Software* **38**, 130–132, 10.1109/MS.2021.3089730 (2021).

[32] Piel, W. H. & Vos, R. A. TreeBASEdmp: A toolkit for phyloinformatic research. *bioRxiv*, 399030 10.1101/399030 (2018).

[33] Wu, P. & Wu, H. *Science Data Bank*10.57760/sciencedb.23014 (2025).

[34] Wu, P. & Wu, H. *Science Data Bank*10.57760/sciencedb.23017 (2025).

[35] Toussaint, E. F. A. *et al*. Explosive Cenozoic radiation and diversity-dependent diversification dynamics shaped the evolution of Australian skipper butterflies. *Evo. J. Linn. Soc.* **1**, kzac001, 10.1093/evolinnean/kzac001 (2022).

[36] Boesing, A. L., Nichols, E. & Metzger, J. P. Biodiversity extinction thresholds are modulated by matrix type. *Ecography* **41**, 1520–1533, 10.1111/ecog.03365 (2018).

